# **The rupture of smaller counterpart aneurysms in patients with multiple intracranial aneurysms**

**DOI:** 10.1038/s41598-025-21914-6

**Published:** 2025-10-13

**Authors:** Thiemo Florin Dinger, Marvin Darkwah Oppong, Mehdi Chihi, Meltem Gümüs, Laurèl Rauschenbach, Mats Leif Moskopp, Yahya Ahmadipour, Maximilian Schüßler, Yan Li, Karsten Henning Wrede, Philipp René Dammann, Ulrich Sure, Ramazan Jabbarli

**Affiliations:** 1 Department of Neurosurgery and Spine Surgery, Center for Translational Neuroscience and Behavioral Science (C-TNBS), University Hospital of Essen, University of Duisburg-Essen, Hufelandstr. 55, 45122 Essen, Germany; 2 Department of Neurosurgery, Faculty of Medicine, University Hospital Carl Gustav Carus, Technische Universität Dresden, Dresden, Germany; 3 Institute of Physiology, Medical Faculty Carl Gustav Carus, Technische Universität Dresden, Dresden, Germany; 4 Institute for Diagnostic and Interventional Radiology and Neuroradiology, University Hospital Essen, University of Duisburg-Essen (Essen, North Rhine-Westphalia, Essen, Germany

**Keywords:** Multiple intracranial aneurysms, SAH, Rupture of smaller intracranial counterpart aneurysm, Neuro-vascular interactions, Cerebrovascular disorders

## Abstract

In case of multiple (unruptured) intracranial aneurysms (M[U]IA), deciding which intracranial aneurysms (IA) should be treated and which at first can be challenging. The most accepted risk factor in making these decisions is IA size. However, a smaller intracranial counterpart aneurysm (SICA) and not the largest IA in patients with MIA might cause subarachnoid hemorrhage (aSAH). By falsely assessing a SICA as benign and withholding treatment, these patients are put at risk for SICA rupture before treatment. Therefore, there is a paramount need to improve the identification of more rupture-prone SICA, especially regarding the improved accessibility to intracranial imaging leading to increasing incidences of patients with (M)IA. From our institutional observational cohort, containing data of all patients with IA treated between 01/2003 and 06/2016, 285 patients with MIA who were hospitalized for acute aSAH were identified. In 261 patients, the largest of their IA ruptured, and in 24 patients, a SICA ruptured (defined by a size difference of ≥ 2 mm). Different demographic, clinical, laboratory, and radiographic characteristics of patients and IA were collected. Univariate and multivariate binary regression analyses (UVA, MVA) were performed to identify putative risk factors for the rupture of SICA. In the final MVA, the total number of IA (*p* = 0.043; aOR = 1.61) and the intake of multiple antihypertensive drugs (*p* < 0.001; aOR = 3.96) showed a statistically significant association with the ruptured status of SICA. In contrast, smoking (*p* = 0.825), radiographic risk factors (i.e., daughter sack *p* = 0.736, IA irregularities *p* = 0.286, location *p* = 0.665), arterial hypertension (*p* = 0.869), and blood examinations did not show a statistically significant regression with the rupture of SICA. This study found statistically significant putative risk factors to identify IA rupture factors that might overweight IA size in certain situations. Thereby, a subgroup of MIA patients could be identified who require treatment with ≥ 2 antihypertensive agents or have a high number of IA that might benefit from a simultaneous treatment of more than one UIA in a single session. Further studies are needed to verify these results and improve the identification of more rupture-prone SICA in MUIA patients.

**Supplementary Information:**

The online version contains supplementary material available at 10.1038/s41598-025-21914-6.

## Introduction

More recently, increasing evidence is questioning the benignity of small intracranial aneurysms (IA)^1–4^. These data are to some extent contradictory to early large natural course studies and established rupture risk scores (e.g., PHASES & UIATS), where, for example, a smaller size (< 7 mm) of anterior circulation IA is regarded as an argument in favor of conservative therapy^5–7^. Together with an aging western population and the increased accessibility to magnetic resonance imaging (MRI), the incidence of (small/multiple) unruptured IA will rise. Patients with small unruptured IA and their treating/consulting neurovascular physicians require clarification on the ambiguous data situation. This need is even more evident regarding the risk rates of 1–4% for serious treatment complications/mortality^8^ and the nearly unchanged, high morbidity and mortality of acute aneurysmal subarachnoid hemorrhage (aSAH) together with its significant socio-economic burden^9^. Even though some studies have identified risk factors and developed risk scores for small IA rupture, more insight is needed to offer valid treatment recommendations^1,3,4^. In particular, patients with multiple intracranial aneurysms (MIA) and their physicians often encounter the challenging decision of which aneurysms should be prioritized for treatment and which can be monitored initially. Also, for MIA cases, IA size is still the predominant parameter for opting for an invasive treatment^10^. However, some studies demonstrated that in 20% to 29% of all MIA patients experiencing an aSAH, the hemorrhage was not caused by the largest IA but a smaller intracranial counterpart aneurysm (SICA)^10,11^. By falsely assessing an unruptured SICA as benign and withholding treatment, these patients are put at risk for SICA rupture. To our knowledge, no previous work has studied the subpopulation of ruptured SICA. An analysis of this subpopulation could help identify rupture-prone SICA and unstable singular IA. Additionally, enabling the correct identification of rupture-prone singular IA and SICA would justify setting more aggressive treatment indications, as already proposed by some authors for certain unruptured intracranial aneurysms (UIA) ≤ 4mm^12–15^. This study aimed to identify putative risk factors associated with the rupture of SICA instead of the large IA.

## Materials and methods

All patients with IA confirmed by digital subtraction angiography (DSA) at the University Hospital of Essen, Germany, were enlisted in our institutional retrospective database between January 2003 and June 2016 and included in this observational, retrospective cohort study. All patients with the diagnosis/suspicion of UIA (symptomatic or asymptomatic) or RIA underwent intracranial DSA due to institutional guidelines. The study was performed in accordance with the Declaration of Helsinki, approved by the Institutional Review Board (Institutional Ethical Review Committee, Medical Faculty, University of Duisburg-Essen, registration number: 15-6331-BO), and registered in the German clinical trial registry (DRKS, Unique identifier: DRKS00008749). Informed consent was not needed due to the protected identity of the patients, the retrospective study design, and the severity of the disease according to the ethics committee and national laws.

### Definition of study aims

The study aimed to unravel associations between known and putative IA protective and risk factors and the rupture of SICA in MIA carriers. Therefore, the patients’ data were screened for socio-demographic and radiological characteristics, pre-existing medical conditions, and blood examinations.

### Definition and documentation of (ruptured) (SC)IA

**Fig. 1:**
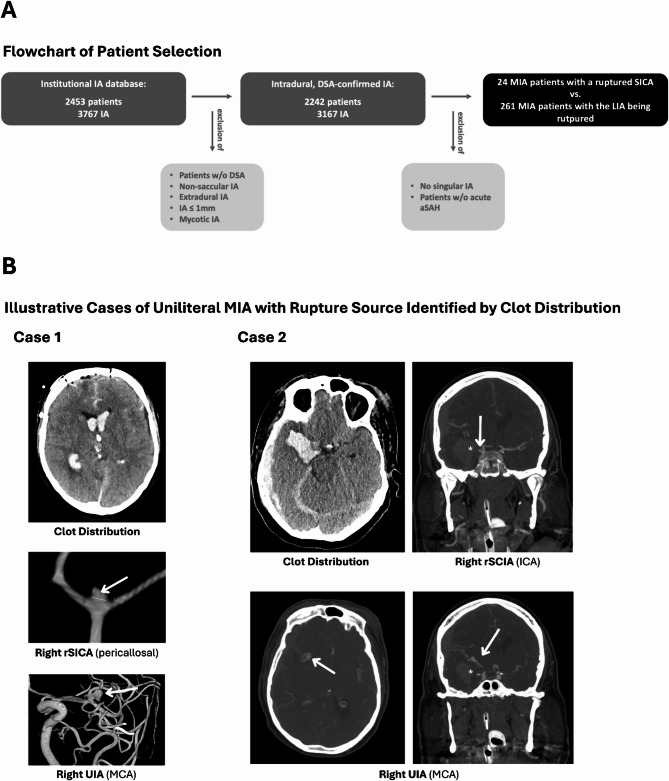
**(A)** This flow chart depicts the selection of patients with multiple intracranial aneurysms (MIA) who suffered an acute aSAH, subdivided into those in whom the largest intracranial aneurysm (LIA) or a smaller intracranial counterpart aneurysm (SICA) was identified as the source of bleeding. **(B)** Two representative cases of unilateral MIA in which a SICA was confirmed as the rupture source. These cases are presented to illustrate the rationale for identifying the bleeding source in challenging scenarios. In **Case 1**, native CT shows severe intraventricular hemorrhage (IVH). Digital subtraction angiography (DSA) reveals a pericallosal aneurysm (white arrow), whose anatomical location in direct relation to the ventricles explains the IVH pattern, whereas the larger middle cerebral artery (MCA) aneurysm showed no plausible connection to the hemorrhage. In **Case 2**, CT (angiography) demonstrates an intracranial clot (marked with *), directly continuous with a ruptured internal carotid artery (ICA) aneurysm (white arrow), while the larger MCA aneurysm did not exhibit any direct anatomical relation to the clot. Off note, in both cases intraoperative findings confirmed the identified source of bleeding. Abbreviations: CTA – computed tomography angiography; DSA – digital subtraction angiography; IA – intracranial aneurysm(s); ICA – internal carotid artery; IVH – intraventricular hemorrhage; LIA – largest intracranial aneurysm; MCA – middle cerebral artery; MIA – multiple intracranial aneurysms; aSAH – aneurysmal subarachnoid hemorrhage; (r)SICA – (ruptured) smaller intracranial counterpart aneurysm; UIA – unruptured intracranial aneurysm; w/o – without.

All patients with MIA hospitalized for acute aSAH were eligible for study inclusion. The diagnosis of an aSAH was first diagnosed by a computed tomography scan, and all patients had a DSA of the neurocranium afterward for further evaluation and identification of all IA. Two experienced neuroradiologists at our university hospital independently reviewed the DSA images. Altogether, the exclusion criteria were (i) missing DSA confirmation, (ii) mycotic origin, (iii) non-saccular morphology, (iv) extradural location, and (v) IA size ≤ 1 mm. For the final analysis, MIA patients with an aSAH were divided into two groups. The first group consisted of patients with a ruptured SCIA (defined by a size difference of at least 2 mm compared to the largest UIA). The second group consisted of patients in whom the largest IA ruptured (Fig. 1). To achieve the highest possible certainty in identifying the RIA in all cases of MIA, two experienced neuroradiologists and two experienced neurosurgeons reviewed the blood clot distribution in CT and the intraoperative/intraprocedural data in each case. As an example of this assessment process, Fig. 1B presents representative cases of unilateral MIA in which a SICA was confirmed as the rupture source. These cases illustrate the rationale applied in challenging situations where multiple aneurysms on the same side had to be evaluated as potential bleeding sources.

### Data extraction

All patients’ electronic charts were screened for demographic, clinical, and laboratory data. IA sizes, locations, morphologies, and numbers were extracted from DSA data. IA location was subsumed into the following groups: middle cerebral artery (MCA), internal carotid artery (ICA), anterior cerebral artery (ACA), and posterior circulation (PC; including posterior communicating, posterior cerebral, basilar, and vertebral arteries). The size of the IA was defined as the longest axis of the IA sack, measured in DSA.

As previously described in detail^16^, the patients’ records were screened to extract demographic and clinical (imaging, pre-existing medical conditions, ABO blood group, and blood examinations) information as summarized in Tables 1 and 2 & Table S1. Regarding blood examinations, only the results obtained upon admission were considered for analysis. Blood values known to be altered by SAH (electrolytes, blood cells, and their properties, creatine kinase, etc.)^17–22^ were excluded from the analysis of IA rupture predictors and only analyzed to verify the described changes in our study. Anemia was defined for females by a hemoglobin (HB) value < 12.5 mg/dL and males by an HB < 13.5 mg/dL.

**Table 1:** Baseline demographic characteristics, established rupture risk factors, and comorbidities of patients with aneurysmal subarachnoid hemorrhage and multiple intracranial aneurysms, stratified by rupture of either the largest or a smaller counterpart aneurysm. *Abbreviations*: ADPKD: autosomal dominant polycystic kidney disease; AHT: arterial hypertension; FIA: Familial intracranial aneurysms; IA: intracranial aneurysm; WFNS: world federation of neurosurgery grade.

Parameter class	large	small
Putative RF	(*n* = 261)	(*n* = 24)
Socio-Demographic		
Age (years)	54.1 (44–62)	51.5 (45–58)
Female	190 (72.8)	19 (79.2)
Ethnicity (non-Caucasian)	11 (4.2)	1 (4.2)
aSAH Severity		
Fisher Grade	3.45 (3–4)	3.34 (3–4)
WFNS grade	2.94 (2–4)	2.90 (2–4)
Established Risk Factors		
AHT	189 (72.4)	17 (70.8)
FIA	5 (1.9)	0 (0)
Smoker	92 (35.2)	9 (37.5)
Comorbidity		
Adiposity	24 (9.2)	4 (16.7)
ADPKD	6 (2.3)	1 (4.2)
Alcohol abuse	24 (9.2)	2 (8.3)
Anemia	42 (16.1)*	7 (29.2)*
Cardiac diseases	52 (19.9)	2 (8.3)
Chronic inflammation	30 (11.5)	2 (8.3)
Diabetes	26 (10.0)	1 (4.2)
Drug abuse	8 (3.1)	0 (0)
Dyslipidemia	34 (13.0)	6 (25.0)
Gastrointestinal diseases	43 (16.5)	5 (20.8)
Gynecologic diseases	18 (6.9)*	1 (4.2)
Hepatic diseases	20 (7.7)	0 (0.0)
Hyperthyroidism	3 (1.1)	0 (0.0)
Hyperuricaemia	5 (1.9)	0 (0.0)
Hypothyroidism	34 (13.0)	2 (8.3)
Musculoskeletal diseases	50 (19.2)	2 (8.3)
Oncologic disease	29 (11.1)	1 (4.2)
Peripheral arterial diseases	41 (15.7)	4 (16.7)
Pulmonary diseases	32 (12.3)	2 (8.3)
Renal diseases	44 (16.9)	3 (12.5)

Values are shown as number, number (%), or mean (interquartile range).

*Data could not be obtained for all patients.

**Table 2:** Morphological and topographic characteristics of ruptured intracranial aneurysms, stratified by rupture of the largest versus a smaller counterpart aneurysm. *Abbreviations*: ACA: anterior cerebral artery; IA: intracranial aneurysm; ICA: internal carotid artery; MCA: middle cerebral artery; PC: posterior circulation.

Parameter class	large	small
Putative RF	(*n* = 261)	(*n* = 24)
No. IA	2.4 (2–2)	2.8 (2–3)
Size (mm)	8.0 (5–10)	4.2 (2–6)
∆size	5.0 (2–7)	4.1 (2–6)
IA sack Irregularity	127 (48.7)*	7 (38.9)*
Daughter sack	63 (24.1)*	4 (22.2)*
Location		
ACA	82 (31.6)	8 (33.3)
ICA	40 (15.4)	7 (29.2)
MCA	68 (26.2)	5 (20.8)
PC	70 (26.9)	4 (16.7)

Values are shown as number, number (%), or mean (interquartile range).

### Statistical analysis

All statistical analyses were performed on SPSS (version 29.0.0.0; IBM Corporation) and OriginPro 2020 (version 9.9.0.225; OriginLab Corporation). Quantitative variables are summarized as mean with interquartile range (IQR), while qualitative variables are presented as absolute numbers and percentages. All putative predictors were checked for a significant association with the rupture of SICA in MIA patients using univariate analysis (UVA). Binary logistic regression analysis was used to identify a statistically significant association. The acceptance level for a type I error (α) was < 5%. All statistically significant parameters were included in the multivariable binary logistic regression analysis (MVA). Before multivariable modeling, predictors were checked for multicollinearity using correlation matrices and variance inflation factors. No relevant collinearity was observed among the included variables. Missing data were handled by multiple imputation using the fully conditional specification (chained equations) procedure as implemented in SPSS. Five imputations with ten iterations each were performed. The automatic method option was applied, whereby SPSS selects the imputation model according to the measurement level of each variable (predictive mean matching for continuous variables, logistic regression for binary variables, and multinomial or ordinal logistic regression for categorical variables). Estimates were pooled using Rubin’s rules. In addition, an exploratory subgroup analysis was performed in unilateral MIA cases, as attribution of the RIA can be challenging when ipsilateral counterparts are present. To assess whether specific combinations of rSICA and its counterpart location may be prone to misclassification, Fisher’s exact tests were applied to explore potential associations between rSICA location and the location of the largest UIA (Table S2).

## Results

For the final analysis, 24 patients with a ruptured SICA and 261 patients with the largest IA being the cause of aSAH could be included (Fig. 1A). In the ruptured SCIA group, the mean age was 51.5 years, with 79.2% being female. The control group’s mean age was 54.1 years, with 72.8% female patients (Table 1).

### Rupture of SICA – demographic aspects

The rupture of SICA did not correlate with patients’ age (*p* = 0.337, odds ratio [OR] = 0.983) in the UVA (Table 3). Also, the female sex was not associated with ruptured SICA (*p* = 0.501, OR = 1.420, Table 3). Lastly, ethnicity did not show a statistically significant regression with the rupture of SICA (*p* = 0.991, OR = 0.988, Table 3).

**Table 3:** Univariate binary linear regression analysis of putative demographic and clinical risk/protective factors of the rupture of smaller intracranial counterpart aneurysms in patients with multiple intracranial aneurysms. Statistically significant risk/protective factors (RF), the 95% confidence interval (CI) of the odds ratio (OR), and the OR are highlighted in grey. Abbreviations: ACE: angiotensin-converting enzyme; AHT: arterial hypertension; ALT: Alanine transaminase; ASA: acetylsalicylic acid; AST: aspartate transaminase; AT1: angiotensin II type 1 receptor; (t)BIL: total bilirubin; CK: creatine kinase; CRP: c-reactive protein; FIA: Familial intracranial aneurysms; γ-GT: γ-glutamyltransferase; HB: hemoglobin; HCT: hematocrit; IA – intracranial aneurysm; LDH: lactate dehydrogenase; MCH: mean corpuscular/cellular hemoglobin; MCV: mean corpuscular volume; PLT: platelet count; RBC: red blood cells; TP: total protein; WBC: white blood cells; WFNS: world federation of neurosurgery grade.

Parameter class Putative RF	*p*-value	95% CI	OR
Demographic			
Age (years)	0.337	0.95–1.02	0.983
Female	0.501	0.51 − 0.395	1.420
Ethnicity (non-Caucasian)	0.991	0.12-8.00	0.988
Established Risk Factors			
AHT	0.869	0.37–2.32	0.925
FIA	0.999	0.00-/	0.000
Smoker	0.825	0.46–2.62	1.102
Imaging (findings)			
Daughter sack	0.736	0.26–2.59	0.821
Fisher grade	0.606	0.48–1.54	0.856
No. of IA	0.015	1.11–2.53	1.671
Location	0.665	0.71–1.24	0.940
Location PC	0.280	0.18–1.64	0.543
IA size	< 0.001	0.51–0.79	0.631
IA irregularity	0.286	0.22–1.56	0.586
Pre-existing medical conditions			
Adiposity	0.247	0.62–6.25	1.975
ADPKD	0.580	0.21–15.95	1.841
Alcohol abuse	0.888	0.20–4.05	0.898
Anemia	0.073	0.92–7.10	2.550
Cardiac diseases	0.182	0.08–1.60	0.365
Chronic inflammation	0.636	0.16–3.11	0.697
Diabetes	0.370	0.05–3.03	0.393
Drug abuse	0.999	0.00-/	0.000
Dyslipidemia	0.114	0.83-6.00	2.225
Gastrointestinal diseases	0.586	0.47–3.77	1.334
Gynecologic diseases	0.628	0.08–4.72	0.601
Hepatic diseases	0.998	0.00-/	0.000
Hyperthyroidism	0.999	0.00-/	0.000
Hyperuricaemia	0.999	0.00-/	0.000
Hypothyroidism	0.512	0.14–2.70	0.607
Musculoskeletal diseases	0.205	0.09–1.69	0.384
Oncologic disease	0.310	0.05–2.67	0.348
Peripheral arterial diseases	0.902	0.35–3.30	1.073
Pulmonary diseases	0.573	0.15–2.90	0.651
Renal diseases	0.584	0.20–2.47	0.705
Prescribed drugs			
β-blocker	0.378	0.56–4.56	1.601
ACE-inhibitors	0.294	0.63–4.51	1.690
ASA	0.998	0.00-/	0.000
AT1-antagonists	0.004	1.70-16.71	5.329
Calcium-antagonists	0.020	1.21–9.31	3.362
Levothyroxine	0.640	0.16–3.13	0.700
Poly-anti-AHT medication*	< 0.001	2.02–12.68	5.067
Statins	0.370	0.54–5.31	1.689

*Dichotomized for patients who take ≥ 2 antihypertensive medications.

### Rupture of SCIA – imaging results

The mean number of IA per patient with a ruptured SICA was 2.8 with an interquartile range (IQR) from 2 to 3 compared to a mean number of 2.4 IA (IQR: 2–2) for patients in whom the largest IA ruptured (Table 2). The total number of IA differed statistically significantly between the two groups in the UVA (*p* = 0.015, OR: 1.671, Table 3).

**Fig. 2:**
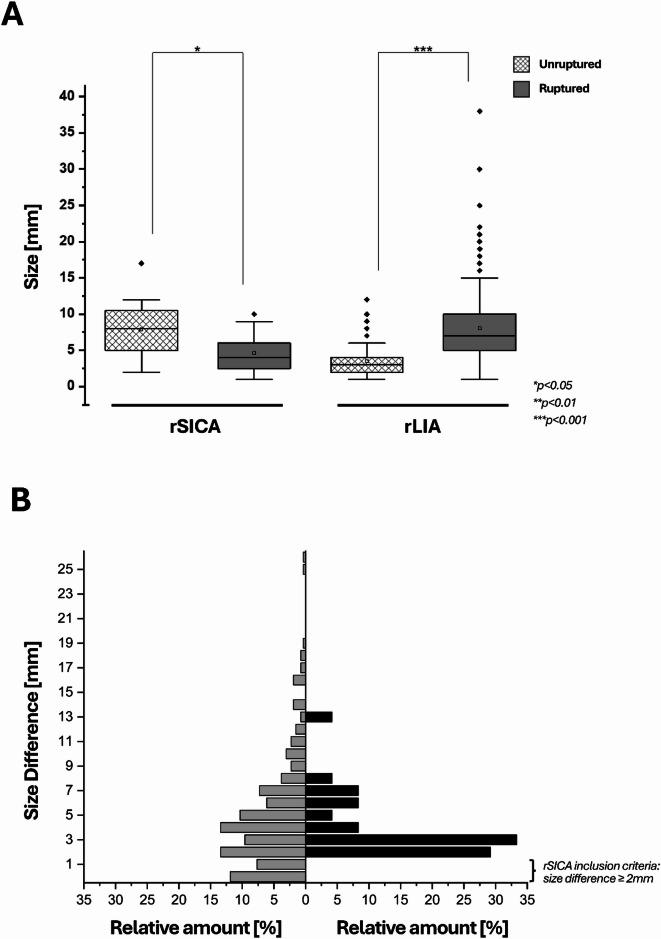
**(A)** Paired boxplots comparing the size [mm] of the largest intracranial aneurysm (unruptured) with the ruptured smaller intracranial counterpart aneurysm (rSICA) on the left side (group rSICA), and on the right side the size of the largest intracranial aneurysm (ruptured, rLIA) with the size of the largest unruptured counterpart aneurysm (group rLIA). In both groups, the size differences between unruptured and ruptured IA were significant (*p* < 0.05), with mean sizes of 4.6 mm (ruptured) vs. 7.9 mm (unruptured) for the rSICA group and 8.0 mm (ruptured) vs. 3.5 mm (unruptured) for the rLIA group. **(B)** Pyramid plot showing the size difference between the ruptured intracranial aneurysm (IA) and its largest unruptured counterpart on the y-axis. The x-axis displays the relative proportion [%] of patients, subdivided into those in whom the largest IA ruptured (rLIA, grey, left side) and those in whom a smaller intracranial counterpart aneurysm ruptured (rSICA, black, right side).

Obviously, the mean size of the ruptured IA (RIA) differed between the two subgroups, with a mean RIA size of 8.0 mm (IQR: 5–10 mm) in case the largest IA ruptured and 4.2 mm (IQR: 2–6 mm) in the ruptured SICA group (*p* < 0.001, OR: 0.631, Tables 2 and 3). The mean size of UIA of the ruptured SICA group (7.8 mm) and ruptured largest IA group (8.0 mm) did not differ significantly (*p* > 0.05; Tables 2 and 3). The size analyses are depicted in Fig. 2A. As demonstrated by this figure, in both subgroups, a statistically significant difference in size could be found between the RIA and largest UIA.

**Fig. 3:**
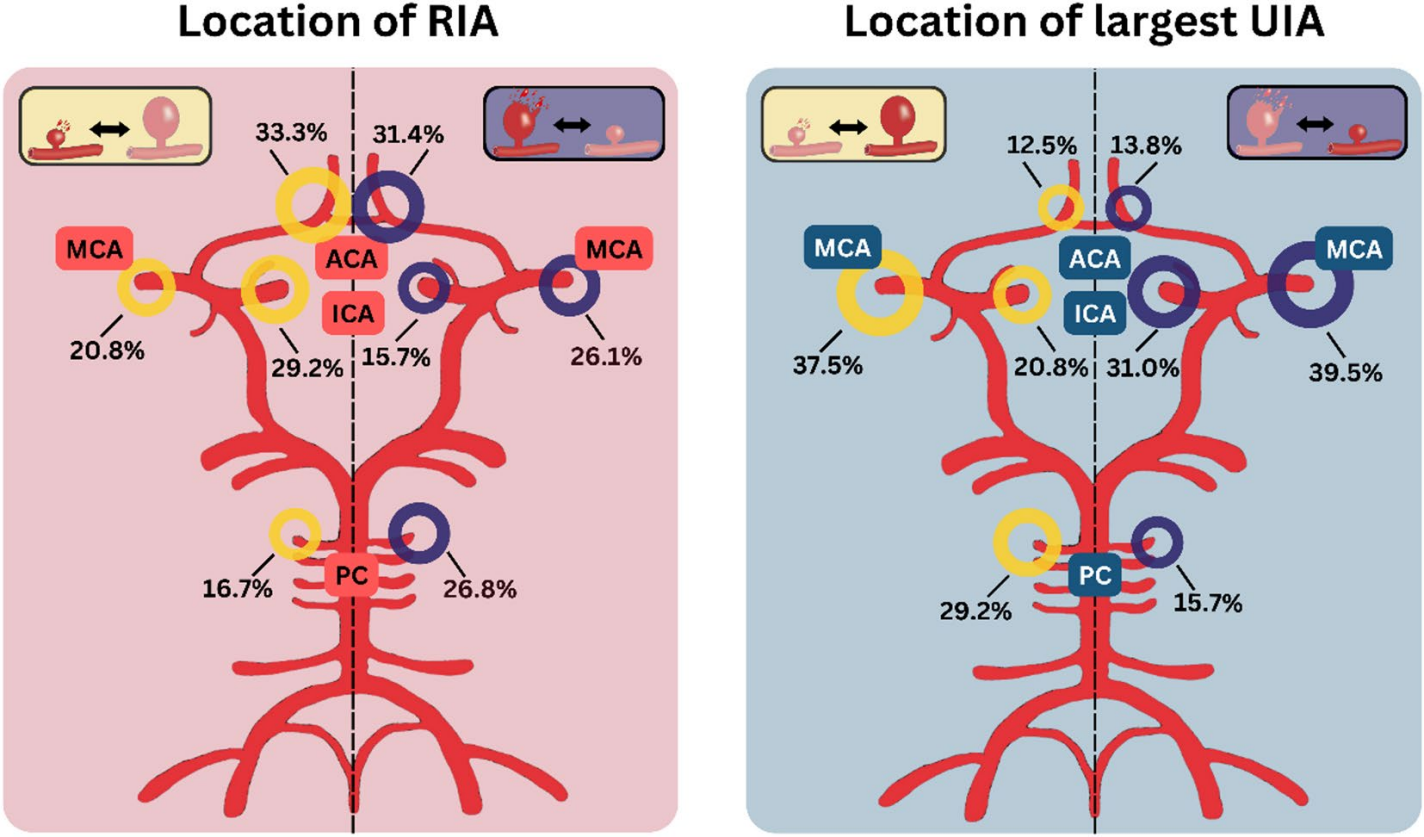
This figure gives a comparison [%] of the location of ruptured (RIA; left side; circle of Willis on red background) and their largest unruptured intracranial counterpart aneurysms (UIA; left side; circle of Willis on blue background). Each circle of Willis is separated in half, demonstrating on the left side the RIA location of patients in whom a SICA ruptured or their unruptured largest intracranial counterpart aneurysms (yellow legend box and rings) and on the right side of the circle of Willis of patients in whom the largest intracranial ruptured or their unruptured largest counterpart aneurysm (purple legend box and rings).

In the next step, the size differences between the RIA and their largest unruptured counterpart aneurysms were analyzed, as shown in Fig. 2B. A mean size difference of 4.1 mm (IQR: 2–6) in the ruptured SICA group and 5.0 mm (IQR: 2–7 mm) in the ruptured largest IA group could be shown (Table 2). All additional imaging parameters, IA irregularities, a daughter sack, or the RIA location did not differ significantly regardless of whether a SICA or the largest IA caused the aSAH (*p* = 0.286, *p* = 0.736, *p* = 0.665, Table 3). Location analyses of the RIA and the largest UIA depicted separately for each group are shown in Fig. 3. Without being statistically significant, we could observe a trend that ruptured SICA are more often located at the ICA than the control group’s RIA (29.2% cp. to 15.7%, respectively, *p* = 0.665, Fig. 3).

### Rupture of SICA – pre-existing medical conditions and medication

Autosomal dominant polycystic kidney disease (ADPKD, *p* = 0.580), arterial hypertension (AHT, *p* = 0.869), diabetes (*p* = 0.370), familiar IA (FIA, *p* = 0.999), and active tobacco consumption (*p* = 0.825) did not show a statistically significant regression with the rupture of SICA in our study (Table 3). Also, for the occurrence of anemia (*p* = 0.073), hyper-/hypothyroidism (*p* = 0.999 and *p* = 0.512, respectively), and renal diseases (*p* = 0.584) no statistically significant regression with the rupture of SICA could be detected (Table 3).

Regarding prescribed medication, the intake of angiotensin II receptor type 1 (AT_1_) antagonists (*p* = 0.004, OR = 5.329), calcium antagonists (*p* = 0.020, OR = 3.362), and more than two antihypertensive agents (*p* < 0.001, OR = 5.067) were significantly associated with the rupture of SICA (Table 3). In contrast, intake of acetylsalicylic acid (ASA), levothyroxine, and statins did not differ significantly between patients with a rSICA and the rupture of the largest IA (Table 3).

### Rupture of SICA – multivariable analysis

The total number of IA and the intake of two or more antihypertensive drugs remained significantly in the MVA (*p* = 0.043 with adjusted [a]OR = 1.610 and *p* = 0.008 with aOR = 3.957, respectively, Table 4).

**Table 4:** Multivariable binary regression analysis of risk/protective factors for the rupture of smaller intracranial counterpart aneurysms (IA) instead of the largest IA in patients with multiple IA. Statistically significant risk/protective factors (RF), the 95% confidence interval (CI) of the adjusted odds ratio (aOR), and the aOR are highlighted in grey. *Abbreviations*: AHT – arterial hypertension; IA – intracranial aneurysms.

Parameter class	*p* value	95% CI	aOR
Putative RF			
No. of IA	< 0.001	1.37–1.94	1.63
Poly-AHT medication*	< 0.001	1.34–1.87	1.58

*Dichotomized for patients who take ≥ 2 antihypertensive medications.

## Discussion

To our knowledge, this is the first study that specifically analyzed the subgroup of MIA patients in whom a SICA ruptured compared to those in whom the large IA ruptured. Previous studies demonstrated that sIA (< 7 mm) are responsible for aSAH in more than half of the cases, and small UIA (≤ 5 mm) of MIA patients seem to have a higher annual rupture risk (0.95%/year) than small singular UIA (0.34%/year)^10,12,23^. Additional work has focused on the rupture of small MIA (< 7 mm)^14,24^. Chen and colleagues used a prediction analysis (decision-analytic Markov model) to compare invasive treatment vs. a “watch and wait” strategy for small MIA. The authors found that endovascular treatment was superior to a conservative treatment^24^. Furthermore, Tong et al. used unsupervised machine learning models to predict the rupture of small unruptured MIA^14^. This group could identify three risk clusters with decreasing rupture risk: (i) patients with a high familiar aSAH burden, (ii) the highest rate of previous aSAH and the highest rate of vascular risk factors, and (iii) no history of previous aSAH and low vascular risk profile.

Following up on the aforementioned studies, the current study identified potential risk factors for rSICA, causing 20–29% of all aSAH in MIA patients, according to the literature^10,11^. The present study revealed that the absolute number of IA and treatment with ≥ 2 antihypertensive agents were statistically significantly associated with the rupture of SICA. The absolute number of IA as a potential risk factor resonates with research exploring systemic influences in aneurysm disease, such as vessel wall vulnerability (“field defect”) and short formation-to-rupture dynamics of small lesions. These perspectives have been described in recent work^11,14,25,26^. While large-scale scores like PHASES did not incorporate multiplicity, likely due to their focus on solitary or larger aneurysms, our results add to ongoing discussions in this field and should be considered exploratory given the small number of rSICA cases^6^. Prior studies have demonstrated the role of AHT as a rupture risk factor in small MIA. Moreover, there is evidence indicating that elevated blood pressure levels or uncontrolled AHT are associated with an increased risk of rupture in patients with IA^12,27^, consistent with biomechanical models suggesting that higher intravascular pressure increases wall stress and rupture probability^28^. In our cohort, treatment with ≥ 2 antihypertensive agents was associated with rupture of SICA. Several hypotheses may account for this observation. One explanation is that the medication reflects insufficient blood pressure control, which would be in line with the hemodynamic models. Another possibility is that class-specific pharmacological effects of antihypertensive drugs, such as anti-inflammatory or vasoprotective actions, influence aneurysm wall stability^29^. These mechanisms are not mutually exclusive and may both contribute to the observed association. A detailed evaluation of these mechanisms, however, lies beyond the scope of this study.

A last crucial finding of our study is that regarding other well-established rupture risk factors (i.e., location, tobacco, female sex, FIA, etc.)^5,30^ no difference could be revealed independently if the large IA or a SICA ruptured. Aneurysm location is nevertheless one of the most established rupture risk factors in the literature, with previous studies consistently demonstrating higher rupture rates for aneurysms located at the anterior communicating artery, posterior communicating artery, or in the posterior circulation^3,6,11,15^. Our finding should not be interpreted as evidence against the general relevance of aneurysm location, but rather indicates that its effect on rupture risk applies similarly to both groups. Based on our data, we suggest that the presence of established MIA, rupture risk factors such as location at the anterior communicating artery, FIA, history of previous aSAH, IA irregularities, and female sex^12–14,24,31^, should direct caretakers also to treat SICA when consulting patients with unruptured MIA who present with a higher number of IA or who require treatment with ≥ 2 antihypertensive agents. In addition, Fig. 2A provides an important implication for treatment strategies. The size difference between the ruptured aneurysm and the largest unruptured counterpart was small in both groups (rSICA: median 4.1 mm; control group: 5.0 mm). This finding underlines that relying on aneurysm size alone may be misleading, as rupture can occur in a smaller intracranial aneurysm of nearly similar size to the largest lesion. In clinical practice, when patients present with multiple aneurysms of comparable size (e.g., a size gap ≤ 3–4 mm), a simultaneous treatment of more than one aneurysm may be considered. This is particularly relevant in patients with a high total aneurysm burden or treatment with ≥ 2 antihypertensive agents, which were independently associated with rSICA in our cohort. Consequently, Fig. 2B highlights that the traditional “largest aneurysm first” approach may not always be sufficient, and that additional clinical and patient-specific factors should guide individualized treatment decisions. These implications are hypothesis-generating and warrant confirmation in prospective studies.

### Limitations

The main limitation of this study is its monocentric, retrospective, and cross-sectional design. The completeness and reliability of data are limited due to the retrospective assessment, and the cross-sectional nature of the study only allows identification of associations but not causal inference or prospective prediction. The study also carries the risk of selection/center bias. Likely, some patients with small UIA have not been referred to our university hospital by general practitioners, outpatient neurologists, neurosurgeons, and radiologists. Additionally, the small number of MIA patients with ruptured SICA could have caused some risk factors to remain undetected in our study. Location (see Fig. 3) as well as morphological surrogates such as irregularity and the presence of a daughter sac were analyzed, whereas more detailed geometric measures were not systematically available in this retrospective dataset. Likewise, clinical variables such as longitudinal blood pressure values were not available, which limited the analysis of systemic factors. Despite the precautions mentioned in the Materials & Methods section, misidentification of the RIA may have occurred in rare cases. Finally, as emphasized by previous studies, the occurrence of rupture in SICA highlights the difficulty of prospectively predicting rupture in this subgroup. It is conceivable that the interval between occurrence and rupture of SICA is short, thereby limiting the opportunity for preventive intervention. Further multicentric, large-scale, prospective studies are needed to validate our results and and to establish their predictive value for clinical decision-making.

## Conclusions

This study found statistically significant putative risk factors to identify IA rupture factors that might overweight IA size in certain situations. Thereby, a subgroup of MIA patients could be identified who require treatment with ≥ 2 antihypertensive agents or have a high number of IA that might benefit from a simultaneous treatment of more than one UIA in a single session to prevent the rupture of SICA. Further studies are needed to verify these results and improve the identification of rupture-prone SICA.

## Supplementary Information

Below is the link to the electronic supplementary material.

Supplementary Material 1

## Data Availability

Any data not published within the article will be shared in an anonymized manner by request from any qualified investigator. In such cases, please contact first author T.F.D. or senior author R.J.

## Abbreviations

ACA: Anterior cerebral artery
ADPKD: Autosomal dominant polycystic kidney disease
ASA: Acetylsalicylic acid
AHT: Arterial hypertension
aSAH: Aneurysmal subarachnoid hemorrhage
AT1: Angiotensin-converting enzyme
CRP: C-reactive protein
DSA: Digital subtraction angiography
FIA: Familiar intracranial aneurysms
HB: Hemoglobin
IA: Intracranial aneurysms
ICA: Internal carotid artery
IQR: Interquartile range
MCA: Middle cerebral artery
MIA: Multiple intracranial aneurysms
MRI: Magnetic resonance imaging
MVA: Multivariable analyses
PC: Posterior circulation
RBC: Red blood cells
RIA: Ruptured intracranial aneurysms
(r)SICA: (ruptured) Smaller intracranial counterpart aneurysm
UIA: Unruptured intracranial aneurysms
UVA: Using univariate analysis
WBC: White blood cells
