# Supplementary material for: The rupture of smaller counterpart aneurysms in patients with multiple intracranial aneurysms
Source: Sci Rep. 2025 Oct 13;15:35569. doi: 10.1038/s41598-025-21914-6 (PMC12518502; doi:10.1038/s41598-025-21914-6)
Supplement: Supplementary file 1 — Supplementary Material 1 [file 41598_2025_21914_MOESM1_ESM.docx]

**Table S1**

Medication use and laboratory findings in patients with aneurysmal subarachnoid hemorrhage and multiple intracranial aneurysms, stratified by rupture of the largest versus a smaller counterpart aneurysm.

Abbreviations: ACE: angiotensin-converting enzyme; ALT: alanine transaminase; ASA: acetylsalicylic acid; AST: aspartate transaminase; AT1: angiotensin II type 1 receptor; (t)BIL: total bilirubin; CK: creatine kinase; CRP: c-reactive protein; γ-GT: γ-glutamyltransferase; HB: hemoglobin; HCT: hematocrit; IA: intracranial aneurysm; LDH: Lactate dehydrogenase; MCH: mean corpuscular/cellular hemoglobin; MCV: mean corpuscular volume; PLT: platelet count; RBC: red blood cell count; TP: total protein; WBC: white blood cell counts.

| **Parameter class** | **large** | **small** |
| --- | --- | --- |
| **Putative RF** | **(n=261)** | **(n= 24)** |
|  |  |  |
| **Blood group** |  |  |
| 0 | 74 (37.2)* | 10 (55.6)* |
| A | 92 (46.2)* | 6 (33.3)* |
| B | 24 (12.1)* | 1 (5.6)* |
| AB | 9 (4.5)* | 1 (5.6)* |
|  |  |  |
| **Blood examination** |  |  |
| ALT [U/L] | 30.3 (15-33)* | 25.2 (16-33)* |
| AST [U/L] | 34.0 (18-37)* | 30.8 (23-36)* |
| (t)BIL [mg/dL] | 0.56 (0.40-0.60)* | 0.52 (0.35-0.65)* |
| Calcium [mmol/L] | 2.3 (2.2-2.4)* | 2.3 (2.2-2.3)* |
| Chloride [mmol/L] | 109.0 (105-112)* | 110.4 (105-114)* |
| CK [U/L] | 201.9 (68-167)* | 176.4 (70-168)* |
| Creatinine [mg/dL] | 0.91 (0.80-0.98)* | 1.11 (0.77-0.97)* |
| CRP [mg/dL] | 2.7 (0.5-2.9)* | 5.8 (0.9-6.5)* |
| γ-GT [U/L] | 39.3 (15-47)* | 43.8 (19-72)* |
| HB [mg/dL] | 13.4 (12.3-14.6)* | 12.5 (11.1-14.1)* |
| HCT [%] | 39.1 (36.4-42.3)* | 37.0 (33.9-39.5)* |
| LDH [U/L] | 221.8 (176-247)* | 237.2 (206-258)* |
| MCH [pg] | 30.7 (29.6-31.9)* | 31.1 (29.6-33.0)* |
| MCV [fL] | 89.8 (86.0-93.1)* | 92.7 (88.7-97.7)* |
| Phosphate [mmol/L] | 3.3 (2.7-3.8)* | 3.5 (2.8-3.9)* |
| PLT [/nL] | 236.8 (201-272)* | 263.6 (217-315)* |
| Potassium [mmol/L] | 4.0 (3.7-4.3)* | 4.1 (3.8-4.4)* |
| RBC [/pL] | 4.4 (4.0-4.7)* | 4.0 (3.6-4.4)* |
| Sodium [mmol/L] | 140.2 (138-142)* | 141.2 (137-146)* |
| TP [g/dL] | 6.6 (6.0-7.1)* | 6.4 (6.2-7.0)* |
| Urea [mg/dL] | 14.1 (10-17)* | 13.6 (10-17)* |
| WBC [/nL] | 13.3 (9.7-16.0)* | 12.4 (9.3-16.3)* |
|  |  |  |
| **Prescribed drugs** |  |  |
| β-blocker | 36 (14.1)* | 5 (20.8) |
| ACE-inhibitors | 42 (16.5)* | 6 (25.0) |
| ASA | 18 (8.6)* | 0 (0.0)* |
| AT1-antagonists | 12 (4.7)* | 5 (20.8) |
| Calcium-antagonists | 23 (9.0)* | 6 (25.0) |
| Levothyroxine | 30 (11.5) | 2 (8.3) |
| Statins | 27 (10.6)* | 4 (16.7) |

Unless indicated otherwise, values are shown as number, number (%), or mean (interquartile range).

*Data could not be obtained for all patients.

**Table S2**

Distribution of the location of the largest unruptured intracranial aneurysm (LIA) by the location of the ruptured smaller intracranial counterpart aneurysm (rSICA) in unilateral MIA cases (ipsilateral only; n = 16). Rows indicate rSICA location; columns indicate LIA location. Cells show n (row %). Fisher’s exact test showed no significant association overall (p = 0.59) and in the prespecified subgroup restricted to rSICA in ICA vs. ACA (2×4 table [highlighted in gray]; p = 0.60).

Abbreviations: ACA – anterior cerebral artery; ICA – internal carotid artery; LIA – largest intracranial aneurysm; MCA – middle cerebral artery; MIA – multiple intracranial aneurysms; PC – posterior circulation; rSICA – ruptured smaller intracranial counterpart aneurysm.

| **rSICA \ LIA** | **MCA** | **ICA** | **ACA** | **PC** |
| --- | --- | --- | --- | --- |
| **MCA** | 0 (0.0%) | 0 (0.0%) | 1 (33.3%) | 2 (66.7%) |
| **ICA** | 1 (25.0%) | 1 (25.0%) | 1 (25.0%) | 1 (25.0%) |
| **ACA** | 3 (42.9%) | 0 (0.0%) | 1 (14.3%) | 3 (42.9%) |
| **PC** | 2 (100.0%) | 0 (0.0%) | 0 (0.0%) | 0 (0.0%) |
